# Letters from Far Japan

**Published:** 1892-03-19

**Authors:** Ernest Hart


### Letters from Far Japan.

By Mrs. Ernest Hart.

### III.—Medical Banquets.

Medical banquets are dreary things in England, not that I, of course, have ever been a guest at these hilarious festivals of male medicos, but like Edgar Poe's raven, I have, from up aloft, often seen most of the fun. In the gallery to which the ladies are relegated on these occasions to be half stifled with the fumes of cooking and gas, and refreshed with champagne, I have had with others the privilege and opportunity of counting the bald heads beneath, and listening till after midnight to the efforts at oratory which constitute one of the charms and the tortures of public dinners. Knowing the type, therefore, it was interesting to see how these things are done in Japan.

The medical profession of the cities which we visited vied with each other in their cordial and generous hospitality to my husband, and welcomed him not only as the representative of the powerful British Medical Association, but as a trusted exponent of the national arts, of which all Japanese are justly proud. Many were the charming dinners and entertainments in which we took part, and were treated as honoured guests in a manner truly Japanese.

We are invited to a medical banquet. The jinrikisha men bowl us along at a rapid pace, till we reach a famous tea house, where a crowd of Japanese doctors have already arrived to receive us. On alighting at the doorway we leave our shoes on the steps. Each side of the entrance hall is lined with pretty little musmes (Japanese girls), who, seated on their heels, and dressed in bright soft colours, bow their heads to the ground, and murmuring gently, bid us welcome. Rising then to their feet, they grasp each one of our hands, and with shuffling gait and many laughs and nods they conduct us along the slippery passages to the reception-room. Here we are received by our hosts, and are entertained for some time by watching lady artists paint on satin in water colours; and produce with extreme rapidity delightful impressionist pictures of flowers, birds, and landscapes, which are afterwards presented to us as pleasant mementoes.

All the doctors are also formally introduced by name to Mr. Hart. Dressed in the old Samarai costume, which consists of full-pleated silk trousers like a woman's skirt, and long loose jacket, they bow, pass compliments in English, German, or Japanese. A panel of the wall is then slid aside, and we pass into the dining-room. It is long, perhaps one hundred feet in length, absolutely empty, except for low square cushions ranged against the wall of three sides of the room. We take our seats on these, my husband and I at the head of the room, on trying in vain to sit in comfort on our heels. Unlike the English, the Japanese get over the serious business of speechmaking before dinner. The speeches are made and translated and listened to with profound silence.

Then dinner begins. From the further end of the long room a bevy of musmes is seen approaching. Each bears a tiny lacquer table about a foot square and four inches high, on which are arranged the little dishes or bowls of the first course. Passing noiselessly along the musme drops on her knees, and places the raised tray on the ground before you. Three or four times during dinner this is repeated, each course consisting of a number of dishes of fish, soup, vegetables, and condiments, all cooked *à la japonaise*, and eaten with chop sticks, except the soup, which is drunk out of the lacquer bowls. A Japanese banquet generally consists of five courses of soup and five of fish, with many extras, such as bamboo shoots, lotus seeds, cuttlefish claws, sea-slugs, &c. The fish is excellent, and the soups are very well made, but somehow a Japanese banquet does not satisfy an English appetite. The food is picked at, not eaten; the little dishes are not removed, and presently the floor becomes littered with the disordered remnants of the numerous courses. A banquet is generally begun with raw fish and ends with a baked carp, impossible to eat with chop sticks, so this, with the boxes of sweets, invariably presented, are put into your jinrikisha to take to your hotel, to your despair the next morning as how to dispose of them. Bowls of rice and tea complete the repast.

Dining is not, however, the serious business it is in England. The Japanese do not meet to eat, but eat because they have met, and conversation and amusements form the principal part of a banquet. Conversation need not be held only with your neighbours, for if a man wishes to speak to a friend in another part of the room he quietly slips the paper panel behind him, passes into the verandah, enters the room again, and sits down on the floor before his friend. Exchanging cups is the chief ceremony at a Japanese dinner. Saké, a spirit made from rice, resembling dry sherry, is drunk hot out of tiny lacquer and gold cups throughout dinner; and the musmes, who sit on their heels in the open space of the floor, patiently watch for every opportunity to fill your cup with saké. When a gentleman would exchange cups—which is equivalent to drinking your health—he sits down in front of you and begs the honour. You empty your cup into a bowl of water, have it filled with saké, drink, wash it again, and hand it to your friend; he raises it to his forehead, bows, has it filled, and drinks. As this ceremony has to be gone through a great many times, drinking is often a mere pretence. I have had to exchange cups as many as twenty or thirty times in the course of a banquet. Eating is, however, as I have said, but a small part of the entertainment. We must be amused, and to amuse is the business of the *geishas*, the licensed singing and dancing girls who are attached to every tea-house. In the empty space in the further end of the room, geishas are seated, and continue playing plaintive and monotonous tunes on the goto and samisan—stringed instruments something like the zither and the guitar—and singing in a high nasal tone. But the singers at a Japanese dinner only take the part of the chorus in a Greek play, and they sing the story which dancing girls represent or suggest by a series of gestures or postures. The dancers are splendidly dressed, and their movements are so interesting, so unlike anything seen in Europe, that we watch them with a curious sense of pleasure. Clever conjurers also fill up the time with incomprehensible tricks of sleight-of-hand. As the hours pass, all reserve breaks down. The men gather into little knots to talk, smoke, and play chess; the musmes and geishas do not leave the room; their aim is to amuse and be gay, and among their accomplishments are numerous little games played with the hands, which they teach and practice with merry shouts of laughter to grave and reverend seigneurs sitting on the floor. Thus the evening passes, till at last it is time to go, and conducted again to the outer hall by the pretty chattering girls, we find our own pair of shoes, get into them and into our jinrikisha, and drive home through the long silent streets, speculating on the marked differences between English and Japanese medical banquets, and contrasting sleepily British waiters with Japanese musmes.

---

A curious investigation was made not long ago in Munich as to the effect of excessive beer drinking upon the health of the inhabitants. The result of the enquiries was probably the reverse of what was expected. It was found that the proprietors of wine-rooms are shorter lived than those of beer saloons, whose average of life is nearly fifty-two years.

